# Medical History from the Earliest Times

**Published:** 1893-03-04

**Authors:** E. T. Withington


### MEDICAL HISTORY FROM THE EARLIEST TIMES.

By E. T. WITHINGTON, M.A., M.B. Oxon.

### XLV.—THE REVIVAL OF LEARNING.

THE Renaissance or Age of Reformation has been compared to a tree which puts forth fresh buds and blossoms in spring-time while the withered leaves of the previous autumn still hang thick upon its branches, and the image may very fairly be applied to the changes which then took place in the healing art. Though we date the beginning of a new epoch in this as in other departments of human knowledge from the invention of printing and the revival of Greek in the middle of the fifteenth century, nearly two hundred years elapsed before the new spirit could be said to have become predominant in medicine. The fruits of the physical sciences ripen more slowly than do the more direct products of the intellect, and it was not till a century after Raphael and Luther that the world was ready for Harvey and Galileo. The influences of the age, however, manifested themselves during this interval in many ways, the more important of which will now be briefly considered.

To take first the revival of classical learning. This begins, from the point of view of the medical historian, with the year 1443, when Thomas of Sarzana, afterwards Pope Nicholas V., discovered in the church of St. Ambrose, at Milan, a manuscript of the "De Medicinâ" of Cornelius Celsus, which had disappeared for many centuries. It was one of the first medical books printed, and gave the physicians of the age their earliest opportunity of studying the best side of the Hippocratic medicine undimmed by the medium of imperfect translations. Ten years later came the fall of Constantinople, and the dispersal of Greek teachers and Greek manuscripts throughout Western Europe. Among the scholars and critics who now sprang up on every side physicians hold a prominent place. At Ferrara, Nicholas Leonicenus translated the Aphorisms of Hippocrates, and pointed out the many errors in that oracle of the Middle Ages, the "Natural History" of Pliny the Elder. At Strasburg and Paris, Günther, of Andernach, professor at once of Greek and of Anatomy, translated Galen, Alexander, and Paulus into classical Latin. At Metz, Anutius Foësius devoted forty years of his life to producing a worthy edition of the works of the Father of Medicine. Meanwhile, our own Linacre was studying physic at Padua and Greek at Florence, where he perhaps took from the Platonic Academy established by the Medici the model of that more famous Royal College of Physicians which he himself founded in London (1518). These are but a few of the medical philologists of the age, and it is interesting to notice that representatives of the Teutonic nations, England and Germany, now become the rivals, and even the teachers of the French and Italians, John Kaye (Caius) lecturing on Aristotle at Padua, while Günther of Andernach taught anatomy at Paris.

The substitution of the works of Celsus and Hippocrates for bad translations from Galen and Avicenna was a great advance, and had, as we shall soon see, a marked effect on medical practice. But the revival of learning gave birth to something of yet greater import and of far wider influence—the spirit of criticism, which, beginning with the disputes of grammarians, gradually invaded every department of human thought and action. When not only passages, but whole treatises, hitherto believed to have been written by Galen and Hippocrates, were shown to be doubtful or spurious, it was natural that men should look for some higher criterion of medical truth than imperfect manuscripts, or still more imperfect translations, and we find one of the earliest philological physicians, Nicholas Leonicenus, inquiring "Why has Nature given us our eyes and other senses, unless that we might rely upon ourselves in the search for what is true?"

In all periods of transition three different classes of men usually make their appearance—the sober reformers, who gradually assimilate the new while holding fast whatever is good in the old; the stubborn conservatives, who refuse to adopt or to give up anything; and the revolutionists, whose aim it is to make a clean sweep of everything in order to set up brand new theories and systems of their own. In medical history the first of these classes was represented by the above-mentioned scholars, or "Greeks," as they were called, and by the great anatomists and clinicians who succeeded them; the second was composed of the "Arabists," or adherents of the dominant school, whose chief text-book was the Canon of Avicenna; while the third comprised the mystic, chemical, or Paracelsic physicians, of whom we shall have much to say later on; and the whole of the sixteenth century is occupied by a sort of triangular duel between the three parties. One of its most striking episodes was the great controversy between the Greeks and Arabists on the question of bleeding in pleurisy, or rather pleuro-pneumonia. Peter Brissot, a distinguished French physician, and great admirer of the Greeks, concluded from his study of the ancients, and observation of an epidemic in 1515, that the Arabist rule of bleeding at a distance from the part affected, or "revulsion," as it was called, was contrary to reason, experience, and Hippocrates, and a danger to the human race, and that the only true method was that of "derivation," or venæsection on the same side as the disease. This was made a test between the two schools, and Brissot, though he made some important converts, was condemned by the faculty of Paris, always the seat of conservatism in medicine. He therefore betook himself to Spain and Portugal, ostensibly to study the medical properties of the strange herbs then being brought from the New World. But his controversy followed him, and raged still more fiercely after his death (1522), and the publication of his "Apology," in which he defends the Greeks, and attacks the Arabs, with much eloquence and learning. The adherents of the latter appealed first to the University of Salamanca, and when it decided against them, to the Emperor Charles V., to whom they declared that the derivationists were the Lutherans of medicine; but a relative of the monarch had recently died under Arabist treatment, so he also decided for the Greeks. Meanwhile the Italian physicians attempted to settle the matter by holding a synod on the theological model at Bologna, under the presidency of Pope Clement VII.; but the prestige of the classic learning and of the term "Hippocratic" was alone sufficient to secure the final victory of the Greeks, though the controversy smouldered on till it was extinguished by Harvey's great discovery.

At the close of the fifteenth century the Arabist school was still decidedly predominant, as is shown by the medical curriculum introduced in 1481 at Tübingen, a recently-founded University, which would naturally adopt the system most in vogue. The course extended over three years, and was thus arranged. First year: Mornings, Galen's "Ars Medica"; afternoons, the two first chapters on Fevers, from the Canon of Avicenna. Second year: Mornings, Avicenna, Book I., on anatomy and physiology; afternoons, the ninth book of Rhazes' "Liber Almansoris," or the corresponding part of the Canon. Third year: Mornings, the Aphorisms of Hippocrates; afternoons, Galen "On Internal Diseases," or "On the Regimen of Health." Additional lectures were given on the surgical part of the Canon, on Mesne's "Simple Medicines," and on Constantine's "Viaticum," also a translation from the Arabic. "Every three or four years, in the coldest season after Christmas, the body of an executed criminal shall be dissected (says the statute), if it can be got. All who take part in the dissection shall attend a mass for the subject's soul every morning; they shall take oath not to remove any part of the body; and they shall all attend the remains to the grave." In the above list we find a proportion of six Arabic to three Greek text-books, but by the end of the next century this will be more than reversed, after which the Arabic writers rapidly disappear.

